# P4 Medicine for Heterogeneity of Dry Eye: A Mobile Health-based Digital Cohort Study

**DOI:** 10.14789/jmj.JMJ22-0032-R

**Published:** 2023-01-26

**Authors:** TAKENORI INOMATA, JAEMYOUNG SUNG, ALAN YEE, AKIRA MURAKAMI, YUICHI OKUMURA, KEN NAGINO, KENTA FUJIO, YASUTSUGU AKASAKI, AKIE MIDORIKAWA-INOMATA, ATSUKO EGUCHI, KEIICHI FUJIMOTO, TIANXIANG HUANG, YUKI MOROOKA, MARIA MIURA, HURRAMHON SHOKIROVA, KUNIHIKO HIROSAWA, MIZU OHNO, HIROYUKI KOBAYASHI

**Affiliations:** 1 Juntendo University Graduate School of Medicine, Department of Ophthalmology, Tokyo, Japan; 1 Juntendo University Graduate School of Medicine, Department of Ophthalmology, Tokyo, Japan; 2 Juntendo University Graduate School of Medicine, Department of Digital Medicine, Tokyo, Japan; 2 Juntendo University Graduate School of Medicine, Department of Digital Medicine, Tokyo, Japan; 3 Juntendo University Graduate School of Medicine, Department of Hospital Administration, Tokyo, Japan; 3 Juntendo University Graduate School of Medicine, Department of Hospital Administration, Tokyo, Japan; 4 Juntendo University, AI Incubation Farm, Tokyo, Japan; 4 Juntendo University, AI Incubation Farm, Tokyo, Japan

**Keywords:** dry eye, big data, mobile health, smartphone application, P4 medicine

## Abstract

During the 5^th^ Science, Technology, and Innovation Basic Plan, the Japanese government proposed a novel societal concept -Society 5.0- that promoted a healthcare system characterized by its capability to provide unintrusive, predictive, longitudinal care through the integration of cyber and physical space. The role of Society 5.0 in managing our quality of vision will become more important in the modern digitalized and aging society, both of which are known risk factors for developing dry eye. Dry eye is the most common ocular surface disease encountered in Japan with symptoms including increased dryness, eye discomfort, and decreased visual acuity. Owing to its complexity, implementation of P4 (predictive, preventive, personalized, participatory) medicine in managing dry eye requires a comprehensive understanding of its pathology, as well as a strategy to visualize and stratify its risk factors.

Using DryEyeRhythm^®^, a mobile health (mHealth) smartphone software (app), we established a route to collect holistic medical big data on dry eye, such as the subjective symptoms and lifestyle data for each individual. The studies to date aided in determining the risk factors for severe dry eye, the association between major depressive disorder and dry eye exacerbation, eye drop treatment adherence, app-based stratification algorithms based on symptomology, blink detection biosensoring as a dry eye-related digital phenotype, and effectiveness of app-based dry eye diagnosis support compared to traditional methods. These results contribute to elucidating disease pathophysiology and promoting preventive and effective measures to counteract dry eye through mHealth.

## Introduction

### Healthcare in the Society 5.0 era: P4 Medicine

Society 5.0 is a novel, human-oriented societal vision proposed during the 5th Science, Technology, and Innovation Basic Plan aimed to promote economic development and resolve various social problems through a highly integrated cyber and physical space^1)^. As part of this vision, healthcare is also presumed to go through a major overhaul with an emphasis placed on providing patient- and public-oriented, predictive, and longitudinal care that can be performed in an unintrusive manner within one's daily life. Medicine envisioned in Society 5.0 utilizes a modern concept of medical big data through mobile health (mHealth) and various mobile device-attached sensors, collecting data on patient-based symptomology, biosensor inputs, and multi-omics in a frequent, longitudinal, remote, real-time, and bidirectional fashion. Such new forms of medical big data can subsequently be integrated to the traditional medical big data, such as electronic medical health records and epidemiologic reports, to be analyzed by artificial intelligence (AI) and generate newfound values of P4 (predictive, preventive, personalized, participatory) medicine^2-7)^.

### Healthcare and the COVID-19 pandemic

The declaration of the coronavirus disease 2019 (COVID-19) pandemic by the World Health Organization (WHO) has dramatically affected the healthcare system^8-10)^. Non-contact and non-intrusive examination techniques have been heavily relied on to minimize the spread of the severe acute respiratory syndrome coronavirus 2 during necessary care, resulting in a global transition to incorporate aspects of telemedicine^11)^. Interestingly, this acceptance of telemedicine brought attention to the limitations of traditional medicine, such as unnecessary referrals to specialists, long waiting time, and crowded hospital environments^12)^. Now gaining traction, digital transformation is offering new solutions to current healthcare problems, including simple remote screening assessments, remote monitoring devices, various app features, AI-assisted diagnosis, and drone-assisted drug delivery system. Considering glaucoma management, a comprehensive work-up on visual acuity, intraocular pressures, visual field tests, and dilated fundus exams is typically expected to be performed at a specialized facility. During the COVID-19 pandemic, while offering on-site care for severe and worsening cases of glaucoma, a protocol can be formulated to triage stable and mild severity patients and offer remote monitoring through portable devices, such as smartphones^12)^. Such an approach can be implemented to other chronic diseases, and efforts to determine reliable digital phenotypes for remote monitoring is crucial for digital advancement.

The worldwide movement toward developing and embracing various non-intrusive life-oriented ocular exams and diagnostic devices have been noted in recent literature^3, 4)^. We developed two smartphone apps, each to collect patient-reported outcomes (PRO) on dry eye and hay fever with ongoing analyses on the accrued big data^13-16)^. Other products include an app for visual acuity check, smartphone-attachable slit-lamp microscope, screening app for diabetic retinopathy, and an app for glaucoma evaluation^17-20)^. Considering the change associated with Society 5.0 and COVID-19 pandemic, healthcare appears to be shifting toward predictive, longitudinal care within one's daily life and away from the traditional facility-based care using mHealth as a central catalyst.

### Symptomology of Dry Eye and its Stratification

Considering the continuing digitalization of modern society, the sheer quality and number of visual screen time are increasing, underscoring the visual impact to one's quality of life (QoL)^21)^. Dry eye is a prevalent ocular disease estimated to be affecting 1 billion patients worldwide and 20 million in Japan alone^22-24)^. Additionally, the aging society, combined with the rapid digitalization that occurred during the pandemic is presumed to escalate the incidence of dry eye in the future^23, 25-27)^. Dry eye presents a wide range of subjective symptoms, including ocular dryness, eye discomfort, decreased vision, and generalized fatigue^28-31)^, which demonstrates a high degree of heterogeneity in its symptomology on an individual basis. Dry eye symptoms have been reported to negatively affect one's quality of vision and work productivity, ultimately resulting in financial burden and societal economic loss^32-38)^. However, the mainstay approach to dry eye management revolves around post-facto treatment of symptoms and suppression of further exacerbation; dry eye currently has no cure^39)^. To establish the groundwork to effectively intervene and prevent further damage in a personalized manner, a cross-hierarchical and -sectional approach may be necessary to analyze comprehensive data on each patient and stratify disease-associated risk factors^3, 5)^.

### DryEyeRhythm^Ⓡ^ smartphone application

**Figure 1:**
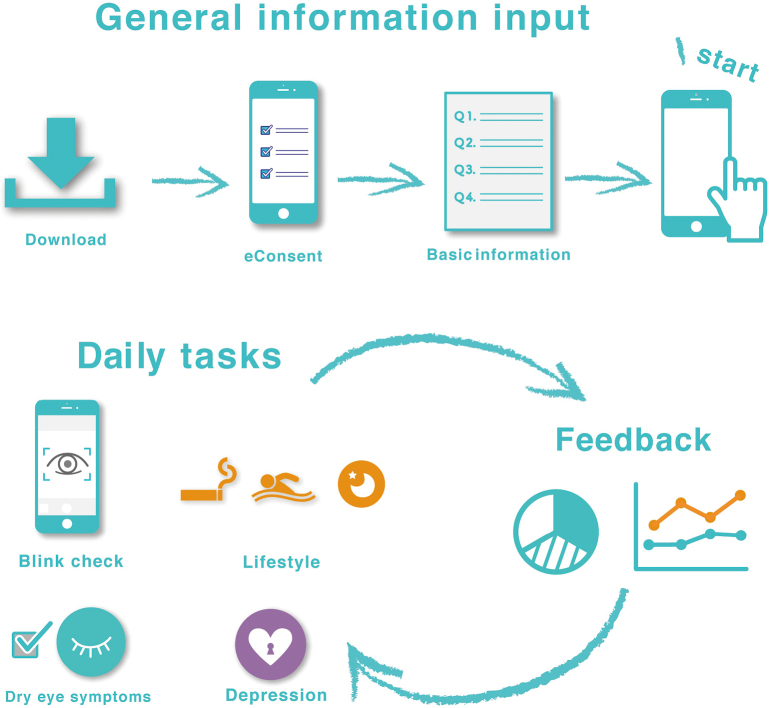
Description of user experience for DryEyeRhythm^Ⓡ^ The figure is used from Inomata T. et al.^6)^ with permission.

**Figure 2:**
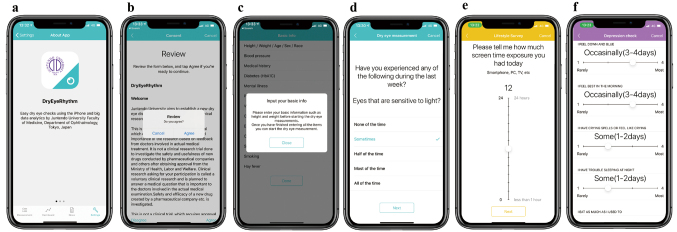
Screenshots of DryEyeRhythm^Ⓡ^ (a)Welcome screen, (b)eConsent, (c) screen for entering participant characteristics, (d)Ocular Surface Disease Index questionnaire, (e) lifestyle information questionnaire, and (f)depressive symptoms questionnaire. The figure is used from Eguchi A. et al.^49)^ with permission.

The DryEyeRhythm^Ⓡ^ app was initially developed using Apple Inc.'s (Cupertino, CA, USA) open-source framework ResearchKit^14)^. This app was released in November 2016 and September 2020 for the iOS and Android versions, respectively, under a consignment contract with the Juntendo University Graduate School of Medicine, Tokyo, Japan, and InnoJin Inc., Tokyo, Japan. It is freely available on Apple's App Store and Google Play. Figure 1 shows the description of the user experience of DryEyeRhythm^Ⓡ^. Following installation of DryEyeRhythm^Ⓡ^ on a mobile device, users are requested to provide electronic consent (eConsent) for participation in the associated research. Table 1 shows the survey items of DryEyeRhythm^Ⓡ^. Upon providing eConsent, users enter their basic information, including age, sex, height, weight, race, educational status, income, and visual acuity, as well as a detailed medical history through the app interface. Users are offered daily tasks to evaluate dry eye-related symptoms, such as a blink sensoring test, answering subjective symptom questionnaires (i.e., Ocular Surface Disease Index questionnaire; OSDI)^28, 40, 41)^, and entering information on the lifestyle patterns. As optional tasks, users can complete an evaluation of the depression symptoms (Zung Self-rating Depres- sion Scale, SDS)^42-44)^ and work productivity (Work Productivity and Activity Impairment Questionnaire)^45)^. Figure 2 shows example screenshots of DryEyeRhythm^Ⓡ^ on a mobile device. The welcome screen of the app can be seen in Figure 2a. Users provided information according to the given instructions, including eConsent (Figure 2b), demographic information (Figure 2c), Japanese version of OSDI (Figure 2d), lifestyle patterns (Figure 2e), and SDS (Figure 2f).

**Table 1:** Survey items of DryEyeRhythm^Ⓡ^

Category	Items
Demographic information	Height, Body weight, Age, Sex, Race, Education, Income, Visual acuity
Medical history	Hypertension, Diabetes, Blood disease, Brain disease, Collagen disease, Heart disease, Kidney disease, Liver disease, Malignant tumor, Respiratory disease, Hay fever, Mental illness including Depression, Schizophrenia, and other than depression and schizophrenia, Past dry eye diagnosis, Ophthalmic surgery including cataract surgery, LASIK, and other than cataract and LASIK, Medication, Eye drop
Lifestyle habits	Coffee, Contact lens use, Screen exposure time, Periodic exercise, Sleeping time, Steps, Smoking, Water intake, Feces
Subjective symptoms	Daily subjective symptoms including eye itching, asthenopia, headache, mental fatigue, stiffness and pain of body axis muscles, and stress, Ocular Surface Disease Index, Zung Self-rating Depression Scale, Work Productivity and Activity Impairment Questionnaire
Blink	Blink counts, Maximum blink interval
Functional visual acuity*	Right visual acuity, Left visual acuity
Others	Latitude, Longitude, Temperature, Humidity, Atmospheric pressure, Weather, Step count

LASIK, Laser in Situ Keratomileusis *Latest version of DryEyeRhythm^Ⓡ^ (after released September 1^st^, 2020) exclude functional visual acuity function.

### Mobile health-based digital cohort studies using DryEyeRhythm^Ⓡ^

Table 2 shows the published studies using the DryEyeRhythm^Ⓡ^ app. Seven articles were published between December 11, 2018 to April 25, 2022^6, 14, 30, 46-49)^. The study types included six cross-sectional studies and one observational study. The number of included participants ranged between 82 to 5,265 participants. Published journals included Ophthalmology, JAMA Ophthalmology, Ocular Surface, Journal of Medical Internet Research, Japanese Journal of Ophthalmology, and npj Digital Medicine.

**Table 2:** Published articles using DryEyeRhythm^Ⓡ^

Authors	Publication date	Study type	Sample size	Age, mean (SD) or median (IQR)	Women rate, n (%)	Findings	Journal
Inomata T et al.^14)^	December 11, 2018	Cross-sectional study	5,265	27.2 (12.4)	3,500 (66.5)	This study identified the risk factors for severe dry eye.	*Ophthalmology*
Inomata T et al.^47)^	January 1, 2020	Cross-sectional study	4,454	27.9 (12.6)	2,972 (66.7)	This study identified the risk factors for symptomatic dry eye and undiagnosed dry eye.	*JAMA* *Ophthalmology*
Inomata T et al.^46)^	April 18, 2020	Cross-sectional study	4,454	27.9 (12.6)	2,972 (66.7)	This study identified the association between dry eye and depressive symptoms.	*Ocular Surface*
Inomata T et al.^48)^	June 26, 2020	Cross-sectional study	4,454	27.9 (12.6)	2,972 (66.7)	This study identified and stratified the individuals with contact lens-associated dry eye and its risk factors.	*Journal of Medical Internet Research*
Eguchi A et al.^49)^	January 8, 2021	Cross-sectional study	2,619	26 (19-40)	1,701 (64.9)	This study determined the eye drop type and usage frequency and identified risk factors for no eye drop use in individuals with symptomatic dry eye in Japan.	*Japanese Journal* *of Ophthalmology*
Inomata T et al.^6)^	December 20, 2021	Cross-sectional study	3,593	27 (20-41)	2,147 (59.8)	This study developed a novel smartphone-based digital phenotyping to stratify heterogeneous symptoms of dry eye into seven clusters and identified the specific profiles and risk factors in each cluster.	*npj Digital* *Medicine*
Okumura Y et al.^30)^	April 25, 2022	Observational study	82	37.4 (11.0)	35 (42.7)	This study determined the reliability, validity, and feasibility of the DryEyeRhythm^Ⓡ^ app for the diagnosis assistance of dry eye.	*Ocular Surface*

SD, standard deviation; IQR, interquartile range

### Risk factors for severe dry eye

Upon conducting a large-scale crowd sourced clinical research using DryEyeRhythm^Ⓡ^, we were able to collect comprehensive, individualized medical big data on dry eye, and identify the risk factors associated with dry eye symptom exacerbations^14)^. DryEyeRhythm^Ⓡ^ was downloaded 18,225 times between November 2016 and November 2017. Odds ratios (95% confidence interval) of each user-reported factors on developing severe dry eye subjective symptoms were calculated for 5,265 users who provided basic information, medical history, lifestyle patterns, and OSDI. Dry eye symptoms were considered severe if the reported ODSI total score was above or equal to 33 points^28, 40)^. Identified factors included younger age by 1 year, 0.99 (0.98-0.99); female sex, 1.85 (1.60-2.14); collagen disease, 2.81(1.34-5.90); depression, 1.68 (1.23-2.29); current contact lens (CL) use, 1.24 (1.09-1.41); hay fever, 1.18 (1.04-1.33); higher on-screen time by 1 hour, 1.02 (1.01-1.03); and smoking, 1.53 (1.31-1.79). Of the identified factors, current CL use, on-screen time, and smoking are modifiable risk factors that may be helpful when advising patients on lifestyle pattern adjustments to prevent dry eye exacerbation. While the identified factors from this study were largely referenced by various previous epidemiological studies^22, 50)^, the advantage of an mHealth approach lies in the comparable results attained by analyzing big data collected from a single, large-scale crowd sourced clinical study. These results highlight the validity of incorporating mHealth principles in clinical studies and may possess implications on the future direction of research methodologies.

### Characteristics and risk factors for undiagnosed symptomatic dry eye

Using the data generated by Japanese users who downloaded DryEyeRhythm^Ⓡ^ between November 2016 to January 2018, the characteristics of undiagnosed dry eye was evaluated. A total of 21,394 discrete, individualized data was generated during this time^47)^. Among the 4,454 participants included in the study, 53.8% (2,395 users) reported dry eye symptoms without a formal diagnosis of dry eye. Risk factors associated with undiagnosed dry eye included younger age by 1 year, 0.99 (0.987-0.999); male sex, 1.99 (1.61-2.46); collagen disease, 0.23 (0.09-0.60); mental illnesses other than depression and schizophrenia, 0.50 (0.36-0.69); ophthalmic surgery other than cataract and laser in-situ keratomileusis (LASIK), 0.41 (0.27-0.64); current CL use, 0.64 (0.54-0.77); and history of CL use, 0.45 (0.34-0.58). Presence of these risk factors can elevate the clinicians' suspicion for an undiagnosed dry eye, aiding in prompt prevention and effective management through risk factor modification.

### Association between dry eye and depression symptoms

**Figure 3:**
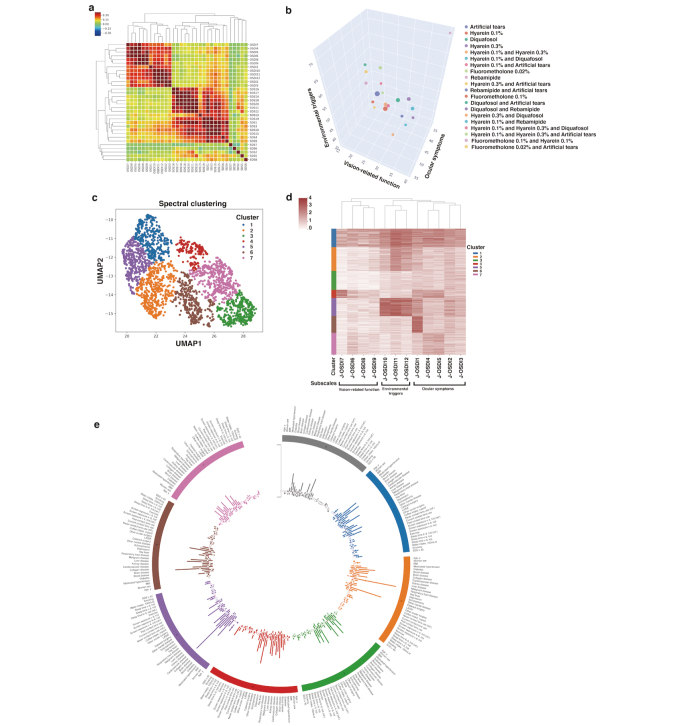
Characteristic visualization of symptomatic dry eye using collected comprehensive dry eye related health data and biosensoring data by DryEyeRhytm^Ⓡ^ (a)The heatmap of the correlation between each item of the Ocular Surface Disease Index (OSDI) and Self-rating Depression Scale (SDS) questionnaires. (b)A bubble chart of representative combinations of types of eye drops used by symptomatic dry eye individuals based on data from the subscales of the OSDI. Among the total 51 combinations, the top 20 eye drop combinations are shown. The X-axis represents the ocular symptoms score, the Y-axis represents the vision related-function score, and the Z-axis represents the environmental triggers score based on the OSDI questionnaire. The bubbles represent the proportion of the combinations of eye drops used. (c)Dimension reduction of individuals with symptomatic dry eye─via Uniform Manifold Approximation and Projection with spectral clustering identified by unsupervised clustering analysis (n=2,619 individuals collected by DryEyeRhythm^Ⓡ^)─depicted seven clusters when stratified for subjective symptoms based on the 12 items of the Japanese version of OSDI. (d)Fraction of individuals within each cluster visualized on the left most panel, along with a corresponding heat map of subjective symptoms from individuals within the identified clusters. (e)Risk factors for each cluster in symptomatic dry eye compared with other clusters visualized in a circular layout. The figures are used from a; Inomata T et al.^46)^, b; Eguchi A. et al.^49)^, and c-e; Inomata T. et al.^6)^, with permission.

The association between mental health and QoL has been gaining attention in recent years, and reports suggest that dry eye shares numerous risk factors with depression, such as disruption of hormonal, metabolic, and neurologic balance, resulting in an increased likelihood of comorbidity^22,51-53)^. Upon examining the connection between the symptoms of depression and dry eye, the results demonstrated that increasing dry eye severity is associated with symptoms of depression^46)^. Notably, the results of this study demonstrated an increased likelihood (3.29 times) of developing depressive symptoms in patients with severe dry eye compared to healthy controls. A hierarchical clustering heatmap of the respective 12 and 20 items of OSDI and SDS visualized the association between each of the item pairs of dry eye and depression questionnaires (Figure 3a). In particular, the environmental factors of dry eye subjective symptoms (OSDI items 10, 11, 12) correlated with the depressive symptoms. With continuous monitoring of the dry eye symptoms, suspicion for comorbid depressive symptoms can be raised upon identification of patients with severe dry eye and interventions can be promptly deployed to prevent or treat dry eye-associated depression.

### Survey of eye drop type, usage frequency, and risk factors of no eye drop use for individuals with symptomatic dry eye

Timely intervention and management through topical eye drops is crucial for preventing dry eye progression^54)^. Of the 2,619 individuals with symptomatic dry eye in this study, 1,876 individuals reported no usage of topical drops^49)^. The most common eye drops used among the individuals were artificial tears (53.4%), hyaluronic acid 0.1% (33.1%), and diquafosol sodium 3% (18.7%). Figure 3b represents a visualized map of various combinations of eye drops used by individuals. The results also revealed that a significant portion of individuals with symptomatic dry eye were not using the recommended dosage of topical drops, raising concerns of decreased adherence. Risk factors (the odds ratio [95% confidence interval]) for no eye drop use were younger age by 1 year, 0.97 (0.97-0.98); body mass index, 1.04 (1.01-1.07); brain disease, 0.38 (0.15-0.98); collagen disease, 0.30 (0.13-0.68); mental illness other than depression and schizophrenia, 0.65 (0.45-0.93); cataract surgery, 0.12 (0.02-0.59); ophthalmic surgery other than cataract and LASIK, 0.55 (0.34-0.88); current CL use, 0.47 (0.38-0.57); history of CL use, 0.58 (0.43-0.77); >8 h on-screen time, 1.38 (1.05-1.81); <6 h sleep time, 1.24 (1.01-1.52); >9 h sleep time, 1.34 (1.04-1.72); and water intake, 0.97 (0.94-0.98). Monitoring dry eye symptoms and medication administration could aid clinicians in accurately understanding the effects of the medications and selecting effective personalized treatment strategies by escalating the intervention when appropriate. In addition, clinicians can more effectively initiate a motivational discussion with the patient to promote and ensure treatment adherence, rather than preemptive treatment escalation.

### Stratifying heterogenous symptomologies and using digital phenotypes for P4 medicine

Dry eye is a multifactorial disease, in which a complex interaction exists between environmental, host, and lifestyle factors that ultimately affects the disease onset, progression, and prognosis^22)^. Therefore, preventive and predictive measures to halt the onset altogether, as well as personalized or stratified approaches for intervention based on each patient's risk factors are considered ideal^3)^. For such strategies, a comprehensive individualized dataset must be first accrued, followed by a cross-hierarchical and cross-sectional analysis. This may elucidate a better understanding behind dry eye symptom heterogeneity, providing a foundation for treatment stratification based on personalized factors^3)^.

**Figure 4:**
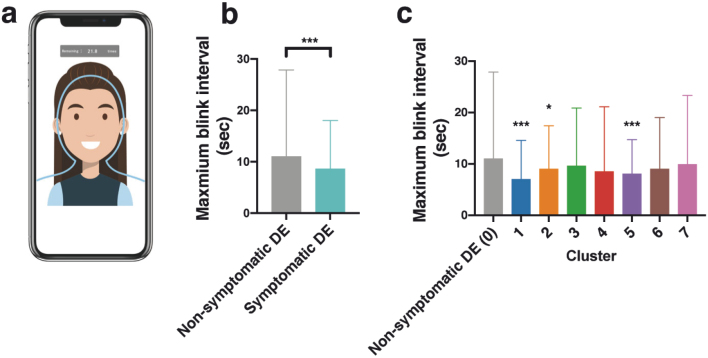
Blink sensoring by DryEyeRhythm^Ⓡ^ (a)The duration of the participant's maximum blink interval (MBI) was recorded by DryEyeRhythm^Ⓡ^. (b)MBI was significantly shortened in symptomatic dry eye vs. non-symptomatic dry eye (8.6 s vs. 11.0 s, ***P<0.001) (c)MBI each cluster (Kruskal-Wallis test, n=3,593, *P=0.016, ***P<0.001). DE, dry eye. The figure is used from Inomata T. et al.^6)^ with permission.

In an effort to combine bioinformatics and AI technology for dry eye research, we developed a smartphone app, DryEyeRhythm^Ⓡ^, which can generate comprehensive medical big data on dry eye through smartphone-attached biosensors and user-provided information^6)^. A total of 3,593 participants who provided consent between November 2^nd^, 2016 and September 30^th^, 2019 were included in the study. The Uniform Manifold Approximation and Projection (UMAP) algorithm was utilized for dimension reduction of the heterogeneous dry eye symptoms, yielding 7 unique subgroups based on the collection of subjective symptoms (Figure 3c). Subsequent visualization of the characteristics of each subgroup was performed through hierarchical clustering (Figure 3d) or a multivariate logistic regression analysis (Figure 3e). Additionally, the performance of maximum blink interval (MBI)^55, 56)^ as a digital phenotype was tested for each identified subgroup using the blink biosensoring feature of the DryEyeRhythm^Ⓡ^ app (Figure 4a). In the subgroups with dry eye, a decrease in the app-measured MBI was observed (Figure 4b). Furthermore, each stratified subgroup displayed distinct MBI changes (Figure 4c). This suggests that the detection of blinking patterns through smartphone apps could be valuable in dry eye screening, as well as predicting the disease subtype.

In this study, dry eye was stratified into unique subtypes that elucidated a pattern based on the symptom heterogeneity, and a novel digital phenotype was identified. Based on the dry eye subtype identified by the DryEyeRhythm^Ⓡ^ app, personalized regimens to mitigate unique subtype-associated risk factors could be possible and clinicians may be able to provide a tailored preventive and interventional plan. This highlights the potential role of mHealth and the generated medical big data in creating a personalized health profile related to dry eye, including symptoms and lifestyle patterns, which have strong implications in bringing principles of P4 medicine to the field of dry eye management.

### Diagnostic performance of dry eye mHealth app

To determine the diagnostic capabilities of DryEyeRhythm^Ⓡ^ through electronic subjective symptom questionnaires and digital phenotyping, the app-based OSDI results and MBI (Figure 4a) measurements were collected from 82 participants above the age of 20 years who visited the Juntendo University Hospital-associated ophthalmology clinic between July 2020 and May 2021. True diagnosis of dry eye was determined using the criteria proposed by the Asia Dry Eye Society, which was used to evaluate the diagnostic performance of DryEyeRhythm^Ⓡ^^57)^. The area under the curve of the resultant receiver operating characteristic curve was 0.910. The sensitivity and specificity of DryEyeRhythm^Ⓡ^ was 91.3% and 69.1%, respectively, suggesting that DryEyeRhythm^Ⓡ^ may be an ancillary non-invasive, non-contact tool for dry eye diagnosis.

## Discussion

Considering the advancements toward healthcare envisioned in the Society 5.0 plan, efforts have been placed to create a medical system that enables ubiquitous care that allows patients and the public to receive non-intrusive care within one's daily life. To realize the framework and improve the quality of dry eye management, the disease heterogeneity and multifactorial nature should be better understood along with efforts to implement P4 medicine in patient care. In this review, a series of studies using DryEyeRhythm^Ⓡ^ to collect comprehensive individualized data on dry eye, including subjective symptoms and disease-associated lifestyle patterns, were discussed to further understand dry eye as a pathology and discover means to implement principles of P4 medicine. mHealth apps appear to be well-suited in creating a holistic dataset for each user, utilizing user-provided data and biosensor data through (now common) smartphone sensors (i.e., camera, touchscreen, global positioning system). mHealth has become more accessible for creating robust medical data sets, which provide insights on disease variability and heterogeneity that traditional methodologies have struggled to provide. Ultimately, this may be the beginning of providing personalized prevention plans, interventions, and behavioral modifications.

mHealth has been receiving attention as a novel and effective route of collecting big medical data^5, 7, 58, 59)^. mHealth, as defined by the WHO, refers to the “medical and public health practice supported by mobile devices, such as mobile phones, patient monitoring devices, personal digital assistants, and other wireless devices.^60)^” In modern research, mHealth smartphone apps are being utilized as a complement to traditional research methodologies^3, 4)^. The use of mHealth apps has several advantages: 1) frequent, longitudinal, remote, and real-time data collection, 2) biosensoring through smart device-attached sensors, and 3) bidirectional participatory medicine. If consent for participation is obtained through the app, the added accessibility and outreach through minimizing the need to physically visit a research facility may be beneficial. In addition, depending on how the data is processed and stored, the comprehensive dataset can stay individualized^4, 58, 61)^. By integrating medical and biosensor data collected through mHealth apps using bioinformatics and AI technology, new aspects of disease processes and variability could be further unveiled.

Recent clinical studies on PRO underscored the importance of including subjective perspectives of patients as part of a routine clinical evaluation^62, 63)^. The studies on electronic PRO (ePRO) collected through mHealth apps showed satisfactory reliability and validity^13, 14)^. One common limitation of mHealth apps was the frequent reliance on self-administered questionnaires, which do not sufficiently reflect the clinical findings and could be prone to biases. However, the blink biosensoring technology included in DryEyeRhythm^Ⓡ^ adds an objective clinical finding as part of the dry eye evaluation, augmenting its accuracy as an ancillary diagnostic tool^6, 30)^. The role of various digital user inputs and lifestyle patterns sensed by smart device sensors that translates into digital phenotypes demonstrates potential in stratifying and visualizing one's disease presentation, which helps bring in new values of P4 medicine into its care.

With an ever-evolving form of medical big data, mHealth appears to be an effective method to capture large data sets and perform a data-driven cross-hierarchical, cross-sectional approach that previous methodologies have struggled to perform^5)^. Its application may be extrapolated beyond dry eye to better understand numerous pathologies in an era of integrated cyberspace and physical space. Additionally, the real-time data collection on one's health and lifestyle and analysis with minimal intrusion enables lifelong medical care that allows providers to rapidly provide tailored medical needs for patients and public based on incoming data^6, 7)^. Considering traditional facility-oriented medicine, a paradigm shift toward a life-oriented healthcare is expected. With the recent changes that accompanied the COVID-19 pandemic, the demand for non-intrusive life-oriented medicine is expected to increase globally, and its research will likely build upon the current foundation laid by mHealth to adopt the four key pillars of P4 medicine.

In conclusion, as the aspects of the Society 5.0 plan comes to fruition, forms of lifelong, non-intrusive, and predictive medicine are expected to emerge. Evolving concepts of medical big data collected through mHealth-through smartphones, wearables, and Internet of Medical Things devices- and AI technology may help bring in new values derived from the principles of P4 medicine to the current system. This warrants a societal discussion to identify technology and strategies that could help transform the healthcare system into one that is more effective and beneficial for patients and the public in our progress toward Society 5.0.

## Funding

This work was supported by Novartis Research Grants 2018 (TI), Daiohs Foundation Research Grants 2018 (TI), JST COI Grant Number JPMJCER02WD02 (TI), JSPS KAKENHI Grant Numbers 20KK0207 (TI), 20K23168 (AMI), 21K17311 (AMI), and 21K20998 (AE), and 22K16983 (AE), the OTC Self-Medication Promotion Foundation (TI and YO); UBE INDUSTRIES FOUNDATION (TI), Charitable Trust Fund for Ophthalmic Research in Commemoration of Santen Pharmaceutical's Founder (TI); TERUMO LIFE SCIENCEFOUNDATION, Kondou Kinen Medical Foundation (TI), Nishikawa medical foundation 2020 (TI); and Takeda Science Foundation 2022 (TI). This study was supported by the following: SEED Co., Ltd.; Alcon Japan, Ltd.; Santen Pharmaceutical Co., Ltd.; Rohto Pharmaceutical Co., Ltd.; HOYA Corporation; WAKAMOTO Co., Ltd. The funders had no role in the design or conduct of the study, collection, management, analysis, or interpretation of data, preparation, review, or approval of the manuscript, or the decision to submit the manuscript for publication.

## Author contributions

TI conceived of the concept of this paper and was major contributor in writing the manuscript. JS and AY were the major contributors in writing the manuscript. TI, YO, KN, KF, YA, AMI, AE, and HT contributed to the development of the study protocol and collected the data. TI, YO, KN, and AMI performed the data analysis and data visualization. TI, YO, AMI, and AE performed funding acquisition. All the authors reviewed the advanced the concepts within the paper and drafted the manuscript. All the authors read and approved the final manuscript.

## Conflicts of interest statement

The DryEyeRhythm^Ⓡ^ application was created using Apple's ResearchKit (Cupertino, CA, USA) along with OHAKO, Inc. (Tokyo, Japan) and Medical Logue, Inc. (Tokyo, Japan). TI, YO, and AMI are the owners of InnoJin, Inc. (Tokyo, Japan), which developed DryEyeRhythm^Ⓡ^. TI reported receiving grants from Johnson & Johnson Vision Care, SEED Co., Ltd., Novartis Pharma K.K., and Kowa Company, Ltd., outside the submitted work, as well as personal fees from Santen Pharmaceutical Co., Ltd., and InnoJin, Inc. The remaining authors declare no competing interests.
